# Evaluation of sentinel lymph node localization in malignant melanoma by preoperative semiconductor gamma camera and planar lymphoscintigraphy

**DOI:** 10.1002/acm2.14077

**Published:** 2023-06-26

**Authors:** Isong Assam, Simon P. Dierck, Yi Zhao, Michael Jüptner, Frank‐André Siebert, Maaz Zuhayra, Ulf Lützen

**Affiliations:** ^1^ Department of Radiation Oncology Ortenau Klinikum Offenburg‐Kehl Offenburg Germany; ^2^ UKSH, Campus Kiel Clinic of Anesthesiology and Operative Medicine Kiel Germany; ^3^ UKSH, Campus Kiel Clinic of Nuclear Medicine, Molecular Imaging and Therapy Kiel Germany; ^4^ UKSH, Campus Kiel Clinic of Radiotherapy (Radiooncology) Kiel Germany

**Keywords:** gamma camera, hybrid SPECT/CT, lymphoscintigraphy, sentinel lymph nodes

## Abstract

**Introduction:**

Performing lymphoscintigraphy in a separate room, frees up the conventional gamma camera, coupled with the desire to directly localize sentinel lymph nodes (SLN) in the operating theatre has led to the development of high‐resolution semiconductor‐detector based handheld gamma‐cameras, CrystalCam.

**Methods:**

This work consists of phantom and clinical studies. For the first part, a Jaszczak phantom with hollow spheres of various volumes were filled with the ^99m^Tc and the camera's sensitivity was measured at various distances to assess the possibilities and limitations of the device. The clinical study evaluates the effectiveness of CrystalCam in localizing SLN in 40 consecutive malignant melanoma patients compared to both conventional planar lymphoscintigraphy and hybrid SPECT/CT. SLNs detected by planar lymphoscintigraphy were marked on the patients’ skin using a UV‐marker. CrystalCam images were acquired in another room by another examiner and the SLNs were marked with a felt pen. The detected nodes by both camera systems were evaluated using UV‐lamp and normal light to visualize the UV‐ and felt pen marks respectively. The concordance rate of the SLNs and higher‐echelon nodes localized by both planar scintigraphy and CrystalCam imaging with respect to the total SLNs and higher‐echelon nodes detected by SPECT/CT imaging are compared and statistically analyzed.

**Results:**

The results of the phantom study show a good correlation between activity and count‐rates for all distancesSPECT/CT, CrystalCamm, and planar lymphoscintigraphy detected 69, 58, and 61 SLNs respectively. The concordance rate of 95.65% by the CrystalCam and planar scintigraphy implies both cameras are statistically coequal in preoperative SLN detection of malignant melanoma. For the higher‐echelon nodes, SPECT/CT, planar and CrystalCam imaging systems identified 82, 48, and 13 respectively; thus, CrystalCam was statistically inferior to planar imaging.

**Conclusion:**

The handheld CrystalCam is a reliable instrument for localizing SLNs in surgical centers without an on‐site nuclear medicine department.

## 1 INTRODUCTION

The current standard of care for malignant melanoma and breast cancer patients is surgical excision after effective sentinel lymph nodes (SLN) localization, thus contributing to the development of less‐invasive surgical procedures. SLN is the first node to receive lymphatic drainage from a tumor. SLN mapping and biopsy techniques utilize dye, radioisotope, or both (dual detection).^1^, ^2^ SLN biopsy for patients with malignant melanoma involves: the intra‐dermal and peritumoral injection of small amount of radiotracer (^99m^Tc labeled colloids) under radiological or sonographic guidance for preoperative scintigraphic imaging and intraoperative re‐identification of the SLN using a gamma probe followed by surgical removal of detected SLNs for pathological analysis.^2^, ^3^, ^4^

Preoperative planar lymphoscintigraphy for visualizing lymphatic drainage pattern from the injection site to SLNs does not show the exact anatomical location of the sentinel nodes.^5^ SLN localization is improved via backlighting ^57^Co flood source transmission images which create an outline of the patient's body, without depicting any internal landmarks.^6^ Hybrid SPECT/CT systems improve spatial location and low contrast resolution, thus showing the anatomical location of visualized SLN. SPECT/CT also detect nodes close to the injection site, accurately localize axillary and extra‐axillary nodes, exclude non‐nodal false positive sites of uptake and contamination sites which are very essential in overweight patients for whom the identification of draining nodes by planar scintigraphy has failed.^5^, ^6^, ^7^, ^8^ Conventional gamma cameras are not readily available in some SLN centers or the need to free up time on gamma camera time in a nuclear medicine department, coupled with the desire to have a direct imaging device in the operating theatre; thus, providing more information for the surgeon at the time of operation, has led to the development of high‐resolution portable handheld gamma cameras utilizing either scintillation crystals or semiconductors detectors.^9^, ^10^

This study evaluates the effectiveness of a small field‐of‐view, handheld preoperative semiconductor‐based gamma camera device CrystalCam (Crystal Photonics GmbH, Berlin, Germany) in localizing SLN in malignant melanoma patients compared to both conventional planar lymphoscintigraphy and hybrid SPECT/CT; where hybrid SPECT/CT was considered the gold standard. Although the physical performance evaluation of CrystalCam in accordance with NEMA standard have been reported by Knoll et al.,^11^ this work includes phantom studies aimed at evaluating the camera's sensitivity and resolution for a range of very low activity uptakes (0.034 to 4.432 MBq) at limited counting interval of 3 s only. The *CrystalClearView* tool of the camera which corrects for the count rates of one nuclide falling into the energy window of another nuclide due to overlapping energy windows when imaging two nuclides with photopeak energies close to each other^12^ was also investigated.

## 2 MATERIALS AND METHODS

### 2.1 CrystalCam handheld gamma camera

**FIGURE 1:**
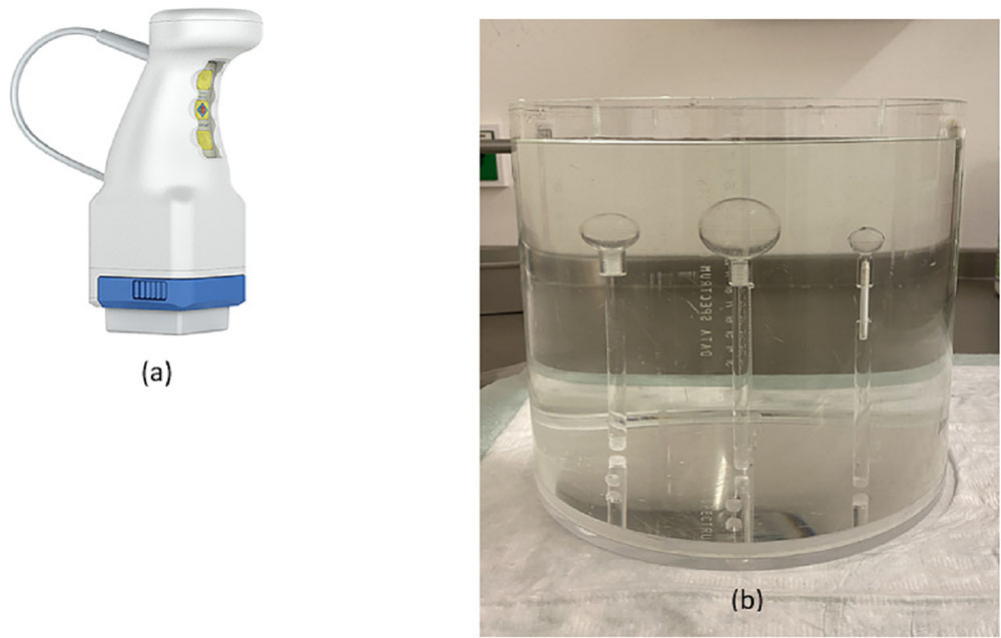
(a) Portable handheld solid‐state gamma camera, CrystalCam (courtesy of Crystal Photonics GmbH, Berlin, Germany) and (b) Jaszczak phantom and hollow spheres mimic the body and “hot” nodes of various sizes respectively.

The handheld gamma camera CrystalCam (CrystalCam) weighing 800 g specifically developed for SLN localization is shown in Figure 1a. The Cadmium‐zinc‐telluride (CZT)‐detector is a 16 × 16 matrix (256 pixels) with a 40 × 40 × 5 mm^3^ dimension and the camera's sides are shielded with 3 mm lead. Four different collimators can be attached to the face of the camera head namely: low energy high sensitivity (LEHS), low energy high resolution (LEHR) collimators, medium energy general purpose (MEGP) collimator which is suitable for energies above 250 keV and the Open Field tungsten collimator with no collimation used for homogeneity calibration, quality control measurements, and transport protection. The collimators can be changed at runtime and are automatically detected by the gamma camera's control and visualization software *Crystal Imager* installed on a standard laptop. The laptop also provides all necessary voltages to run the camera via the USB‐port. The camera system includes a fillable flood field phantom used for calibrations and quality control measurements.^11^, ^12^

### 2.2 Cylindrical Jaszczak phantom and hollow spheres

A cylindrical Jaszczak phantom filled with radioactivity‐free water and hollow spheres with volumes 0.5, 1, 2, 4, 8, and 16 mL were filled with ^99m^Tc. The Jaszczak phantom and the hollow spheres mimic the patient's body and “hot” nodes (0.5 mL) or body organs of various sizes and uptake activities respectively as shown in Figure 1b.

### 2.3 Phantom studies

Knoll et al.^11^have already evaluated the physical performance of CrystalCam in accordance with NEMA guidelines. The phantom studies in this work supplements some physical performance measurements, such as count rate linearity or sensitivity at various source‐detector distance (SDD), the camera's ability to resolve two spheres with activity less than 5 MBq at limited exposure time of 3 s and *CrystalClearView* tool for dual nuclide imaging. Some of the phantom measurements in this work, though not directly relevant for SLN in melanoma, can be useful in other general nuclear medicine imaging situations, aimed at demonstrating the possibilities and limits of the handheld gamma camera system.

#### 2.3.1 Sensitivity measurements

The aim was to investigate how the camera's detected count rates per unit activity or count rate linearity varies with sphere volume and SDD of a low‐contrast hot sphere at a limited counting interval. Each hollow sphere with volumes 0.5, 1, 2, 4, 8, and 16 mL was filled with the same activity concentration of ^99m^Tc, these spheres whose activities are proportional to their volumes were categorized into *Group A* while *Group B* spheres were filled with a calculated activity concentration of ^99m^Tc such that the sphere activities were the same irrespective of sphere volume. Four activity concentrations of 0.068, 0.135, 0.175, and 0.277 MBq/mL were used to produce *Group A* sphere activities ranging from approximately 0.034 to 4.432 MBq. Table 1 shows the sphere volumes and their respective activities when filled with an activity concentration of 0.068 MBq/mL. The 0.5 mL sphere simulates a very low‐contrast node with an uptake of 0.034 MBq.

**TABLE 1:** *Group A*’s sphere volumes and their respective activities prepared by filling each sphere with an activity concentration of 0.068 MBq/mL.

Sphere volume (mL)	0.5	1	2	4	8	16
Sphere activity (MBq)	0.034	0.068	0.136	0.272	0.544	1.088

For *Group B*, four sphere activities of approximately 0.5, 1, 2, and 3 MBq were used. The activity concentrations used in filling the respective spheres to give each sphere an activity of 1.0 MBq is shown in Table 2. Each *Group A* and *B* sphere's activity have a maximum uncertainty of ±0.03 MBq.

**TABLE 2:** Sphere volumes and their respective activity concentrations used to give each sphere an activity of 1.0 MBq.

Sphere volume (mL)	0.5	1	2	4	8	16
Activity Con. (MBq/mL)	2.02	1.01	0.51	0.25	0.13	0.06
Sphere activity (MBq)	1.0	1.0	1.0	1.0	1.0	1.0

**FIGURE 2:**
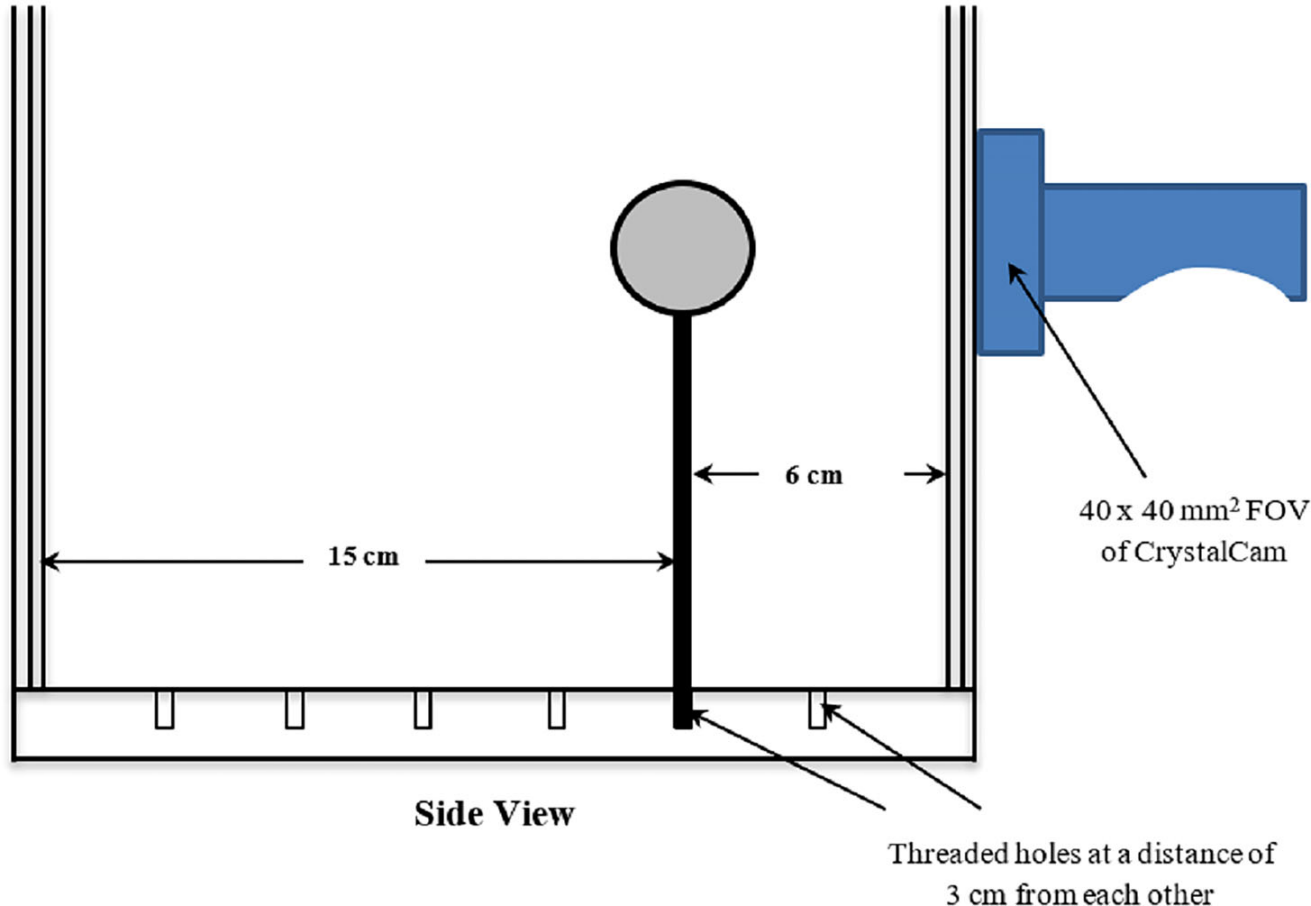
Sensitivity measurements set‐up; sphere filled with ^99m^Tc simulating a “hot” node or region was positioned in the threaded‐hole of the Jaszczak phantom which was filled with non‐radioactive water mimicking the body. At each sphere position, two measurements at two SDDs were acquired by measuring at opposite ends of the phantom, for example, 6 and 15 cm as shown.

Each *Group A* and *B* sphere was positioned in the threaded‐holes drilled at the bottom of the cylindrical Jaszczak phantom filled with non‐radioactive water. The phantom and “hot” sphere mimic the patient's body and “hot” SLN (0.5 mL) or region respectively. Two SDDs were gained per threaded‐hole position by holding the camera at opposite sides of the phantom as shown in Figure 2. Each sphere was measured at SDDs of 3, 6, 9, 12, 15, and 18 cm and the camera was adjusted to the height of the sphere.

After a successful daily quality assurance tests, five measurements of the count rates were conducted per SDD using the LEHS collimator with an acquisition interval of 3 s and an energy window setting of 15% centered on the 140 keV photopeak of ^99m^Tc (129.5–150.5 keV). Each sphere activity was decay‐corrected to compensate for the time difference between sphere activity preparation and measurement. The mean count rates were plotted against the decay‐corrected sphere activities at the particular SDD and their relationship at the respective SDD was statistically analyzed by linear regression using MATLAB (The MathWorks Inc., Natick, MA).

#### 2.3.2 Resolution measurements

**FIGURE 3:**
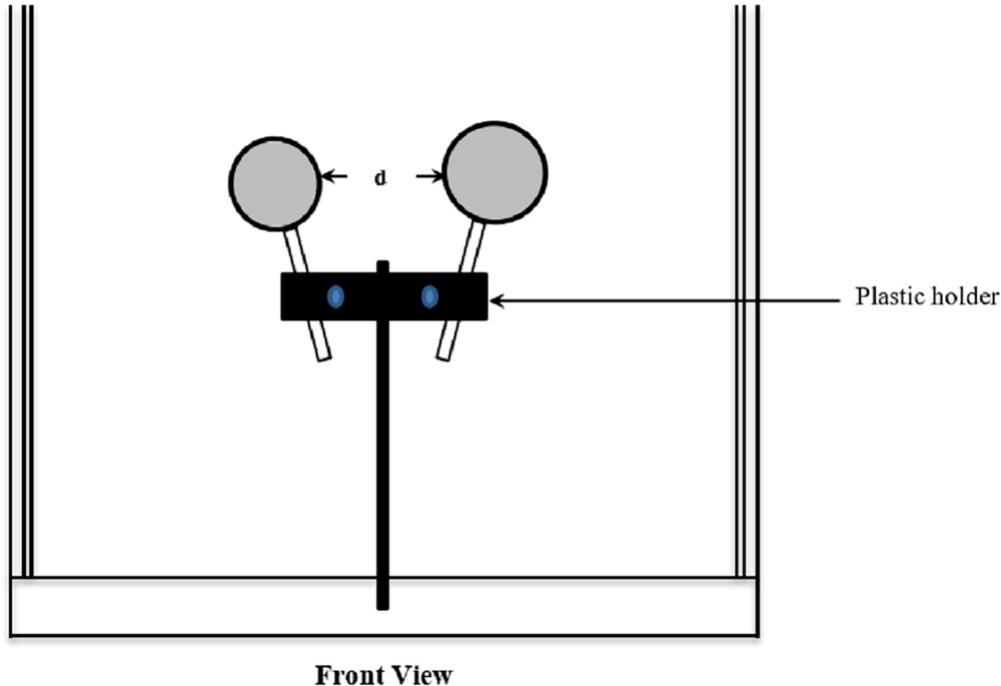
Sphere resolution acquisition setup; the 0.5 and 1 mL spheres filled with ^99m^Tc to simulate “hot” nodes and injection sites respectively at a fixed SDD of 6 cm. Five images were acquired per sphere combination at a distance between the spheres (*d*) of 0, 2.5, 5, 7.5, and 10 mm, where *d* = 0 mm was the case where both spheres were touching each other.

The objective was to investigate CrystalCam's ability to differentiate two hot spots or spheres of different sizes and activities that are close to each other. Two spheres were held horizontally by a plastic holder and imaged for 3 s using LEHR collimator at a fixed SDD of 6 cm, and an inter‐sphere distance (*d*) of 0, 2.5, 5, 7.5, and 10 mm as shown in Figure 3, where *d* = 0 mm was the case where both spheres were touching each other. Only the 0.5 and 1 mL sphere combinations were imaged, where the 0.5 and 1 mL spheres mimic a node and an injection site respectively. It was difficult to image both spheres at a superficial SDD of 3 cm with this setup due to CrystalCam's small FOV.

The 0.5 and 1 mL spheres were filled with 0.75 and 3 MBq of ^99m^Tc respectively, thus simulating an SLN close to an injection site. Two 1 mL spheres filled with 3 MBq of ^99m^Tc each were also imaged with an exposure time of 3 s. To simulate two nodes with the same volume but different uptake activities, two 0.5 mL spheres were filled with 0.75 and 1.5 MBq of ^99m^Tc respectively. For two spheres of different volumes but the same activity, the 0.5 and 1 mL spheres were each filled with 0.5 MBq of ^99m^Tc and these sphere combinations were imaged to investigate the camera's ability to separate two spheres that are close to each other. The resolution measurements are analyzed visually by an observer.

#### 2.3.3 Dual nuclide imaging

The primary objective was to investigate the *CrystalClearView* tool which corrects for the count rates of one nuclide falling into the energy window of another nuclide due to overlapping energy windows when imaging two nuclides with photopeak energies close to each other.^12^ Localized SLNs are marked on the patient's skin with a ^57^Co pen‐point marker not only in this study but in routine clinical practice as well. Investigating the effect of *CrystalClearView* in dual nuclide imaging is paramount due the overlapping energy windows of the ^99m^Tc and ^57^Co.

After a successful camera calibration for ^123^I with LEHS collimator,^11^, ^12^ a 15% energy window was centered on the 140 and 159 keV photopeaks of ^99m^Tc (129.5–150.5 keV) and ^123^I (147.1–170.9 keV) respectively. Two 1 mL spheres each filled with 0.173 MBq of ^99m^Tc and ^123^I respectively, were imaged at an SDD of 6 cm and inter‐sphere distance (*d*) of 5 mm (Figure 2). A 1 mL sphere filled with a mixture of ^99m^Tc (0.06 MBq) and ^123^I (0.11 MBq) to give an activity ratio of 1:2 respectively was also imaged at an SDD of 3 cm (Figure 1). All spheres were imaged with the *CrystalClearView* tool disabled (default) and enabled with an acquisition interval of 3 s. The count rates ratios of both nuclides were compared with their respective sphere activity ratios to evaluate what percentage of count rates of both nuclides was corrected by the *CrystalClearView* tool.

### 2.4 Clinical studies

Lymphoscintigraphies were performed in 40 consecutive patients suffering from malignant melanoma. The patient demography consisted of 18 females and 22 males with a mean age of 62.1 years. Eligibility criteria for this study were as follows: (1) patient must be pathologically diagnosed and histologically confirmed MM from pT1A stage; (2) SLN staging in case of clinical and normal preliminary sonography examinations; (3) all patients must give their informed written consent and be above 20 years. The exclusion criteria were: (1) known hypersensitivity to human albumin derivatives; (2) confirmed metastasis by imaging; (3) clinically high‐grade suspicion of lymphogenic or hematogenic metastasis; (4) pregnancy and lack of mobility.

^99m^Tc ‐nanocolloid with a 23G cannula was injected intradermally and peritumorally by a nuclear medicine physician. The minimum, maximum, and mean injected activities were 31, 90, and 62.70 MBq respectively. In accordance with the EANM guidelines,^13^ dynamic images were acquired immediately after radionuclide administration with a 128 × 128 matrix over 30 min (5 frames à 60 s and 5 frames à 5 min). Routine planar and hybrid SPECT/CT images were acquired using the Symbia Intevo 6 (Siemens, Erlangen, Germany) dual‐head gamma camera with an energy window setting of 20% centered on the ^99m^Tc photopeak (126–154 keV) and the LEHR‐collimators. Static images were acquired using a 512 × 512 matrix over 5 min with a ^57^Co flood source used for body contouring. After the planar imaging, the tip of a ^57^Co pen‐point marker was position directly above the detected SLN such that both the detected SLN and the ^57^Co pen‐point marker was superimposed on each other. This helps to accurately mark the positions of the detected SLNs on the patient's skin with a UV‐marker by a first examiner. The ^57^Co pen‐point marker was imaged with an energy window of 122 keV ± 7.5% (112.9–131.2 keV). Thus, ^99m^Tc and ^57^Co have overlapping energy windows.

The patient was then moved to another room with normal lighting (no UV‐lamp) where lymphoscintigraphy was performed with the handheld gamma camera, CrystalCam, by a second examiner. The possibility of the second examiner being biased was greatly eliminated since the UV‐marker can only be visualized under a UV‐lamp. The CrystalCam's images were acquired with a 15% energy window and an acquisition time of 3 s using the LEHS collimator (phantom studies’ settings). For cases where the injection site or another accumulating lymph node was too close to the measured area, a LEHR collimator was used in addition to LEHS collimator. The occurrence of SLNs in different body regions such as head and neck, axilla, and groin make it technically difficult to hold the handheld camera at a fixed SDD with a camera holder in this study. The hand camera was thus held at arbitrary distances close to the skin. The 3 s acquisition time was the time in which the displayed camera image was automatically refreshed during imaging. The total acquisition time per patient was between 10 to 20 min. The 3 s short exposure time was to avoid high detector deadtime due to the large injection site activity coupled with the small SDD.

The SLN localized with CrystalCam was marked on the patient's skin using a ^57^Co pen‐point marker and the CrystalCam's imaging window was switched from the single ^99m^Tc window to dual ^99m^Tc and ^57^Co isotope windows to confirm that the ^57^Co mark was on the localized SLN as shown in Figure 11b. The ^57^Co mark was accurately duplicated on the patient's skin with a conventional felt pen by the second examiner. The number and location of the detected SLNs and higher‐echelon nodes by both cameras were evaluated by counting the number of UV‐marks and felt pen marks on the patient's skin visualized using a UV‐lamp and normal light respectively. The locations of SLN were categorized under the three body regions; viz. head and neck, axilla, and groin. Lastly, a hybrid SPECT/CT imaging of the relevant regions was performed and the SPECT/CT imaging was considered the gold standard in this study. SLNs that were not localized by SPECT/CT but were either detected by planar imaging or CrystalCam were considered as false positives. Surgical SLN‐biopsy was based on the SPECT/CT and planar imaging and the SLNs were histologically processed. All SPECT/CT localized SLNs were re‐identified by the gamma probe followed by surgical removal.

The McNemar *χ* ^2^‐Test with Edwards correction (*p*‐value < 0.05) and the Cohen's kappa Test were used to statistically compare the total number of nodes (both SLNs and higher‐echelon nodes) localized by both planar scintigraphy and CrystalCam preoperative imaging with respect to the total nodes detected by SPECT/CT imaging.

## 3 RESULTS

### 3.1 Sensitivity measurements

**FIGURE 4:**
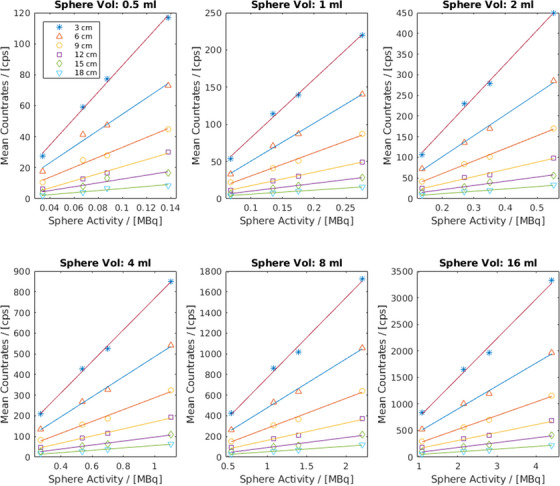
Sensitivity measurement results of *Group A* spheres show that for any given sphere volume and SDD, the mean count rates increase with the sphere's activity.

**FIGURE 5:**
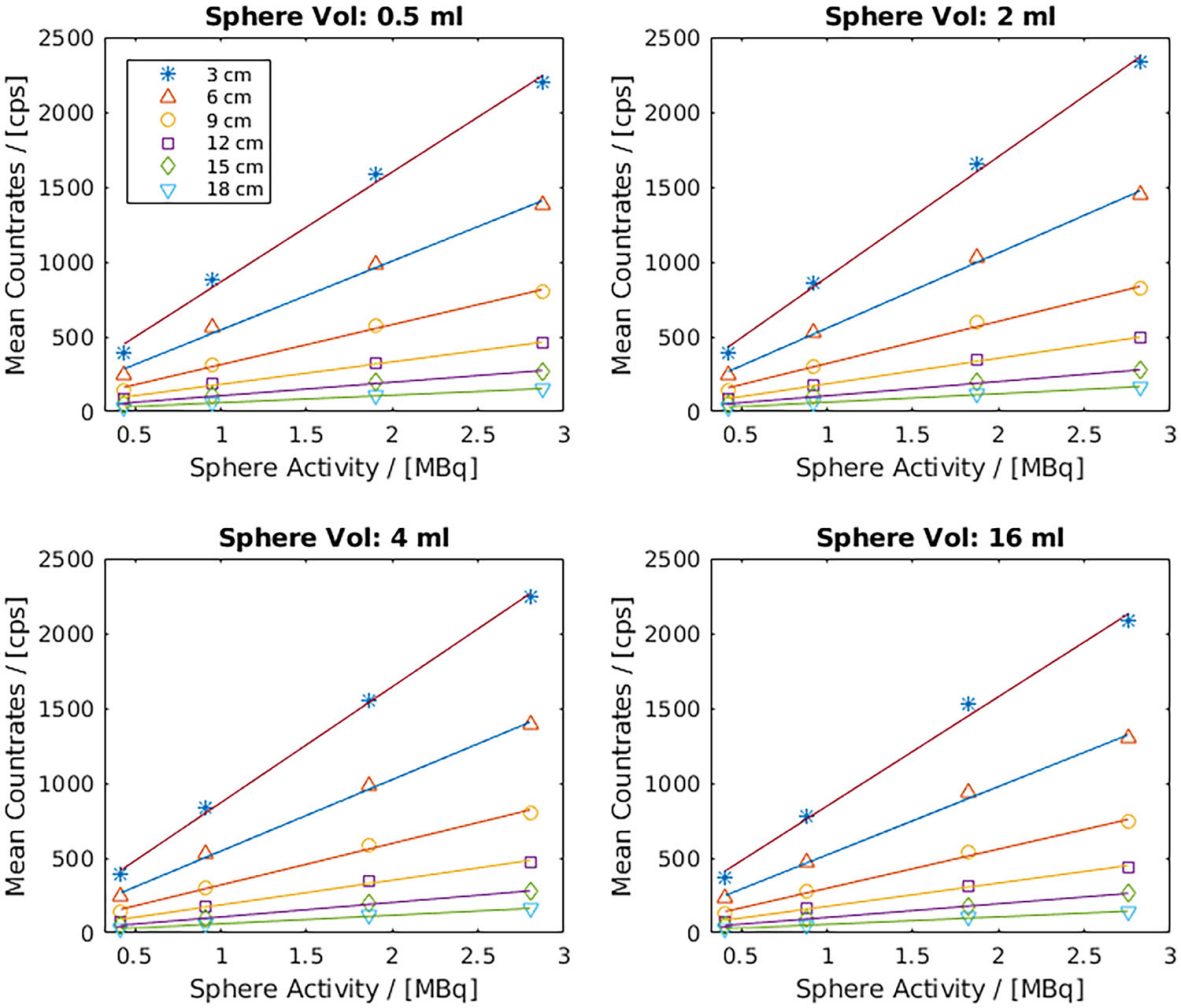
Sensitivity measurement results of *Group B* spheres show a minimum effect of scattering on the measured count rates especially between the 0.5 and 16 mL spheres with an activity concentration ratio of 1:32 at an SDD of 18 cm.

The plots of the mean count rates per SDD measured with CrystalCam and the decay‐corrected sphere activities at the specific SDD for spheres of *Group A* and *Group B* are shown in Figures 4 and 5 respectively. In Figure 4, the sphere activities increase with sphere volumes while spheres in Figure 5 have constant activities irrespective of their volumes thus confirming the accuracy of the activity preparation and filling of spheres. For any given sphere volume and SDD, the mean count rates increase with the sphere's activity.

The 0.5 and 16 mL spheres in Figure 5 have similar mean count rates of 61 and 53 cps respectively for 1 MBq sphere activity at 18 cm SDD, thus affirming a very minimal effect of scattering on the measured count rates in the 16 mL sphere compared to the 0.5 mL despite an activity concentration ratio of 1:32.

The lines on the SDDs plots of Figures 4 and 5 are the linear regression fits of the measurements at the given SDD and the gradient of each line is the sensitivity of the handheld gamma camera at that SDD. The sensitivities (mean ± SD) of all the measurements at the specific SDD and their respective coefficient of determination (*R*2) are shown in Table 3.

**TABLE 3:** The sensitivities and coefficient of determinations (*R* ^2^) at the given source‐detector distance (mean ± SD).

Source‐detector distance (cm)	Sensitivity (cps/MBq)	*R* ^2^
3	785.05 ± 39.97	1.00 ± 0.002
6	486.70 ± 30.84	1.00 ± 0.004
9	290.18 ± 21.69	0.99 ± 0.003
12	174.19 ± 24.50	0.99 ± 0.004
15	99.64 ± 10.83	0.99 ± 0.017
18	55.99 ± 5.13	0.98 ± 0.018

### 3.2 Resolution measurements

**FIGURE 6:**
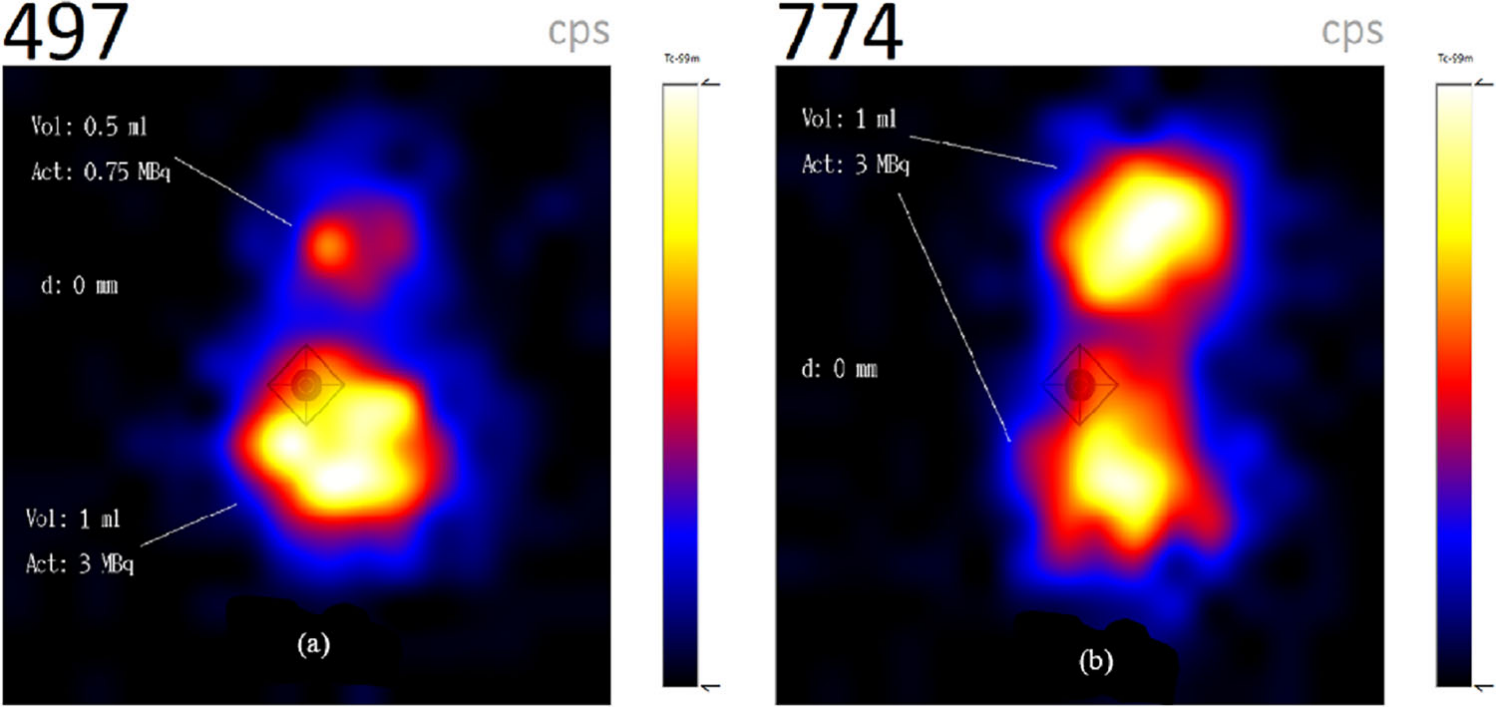
(a) Two spheres mimicking a node close to an injection site and (b) two spheres with the same volume and activity touching each other (*d* = 0 mm).

The sphere combination mimicking the node (0.5 mL) and injection site (1 mL) close to each other is depicted in Figure 6a with the larger sphere brighter with respect to the smaller sphere while Figure 6b shows the two 1 mL spheres with the same activity of 3 MBq having approximately the same brightness. Both spheres in Figure 6a,b are touching each other with an inter‐sphere distance (*d*) of 0 mm. Images with larger inter‐sphere distance (*d*) showing better differentiation are not shown due to space.

**FIGURE 7:**
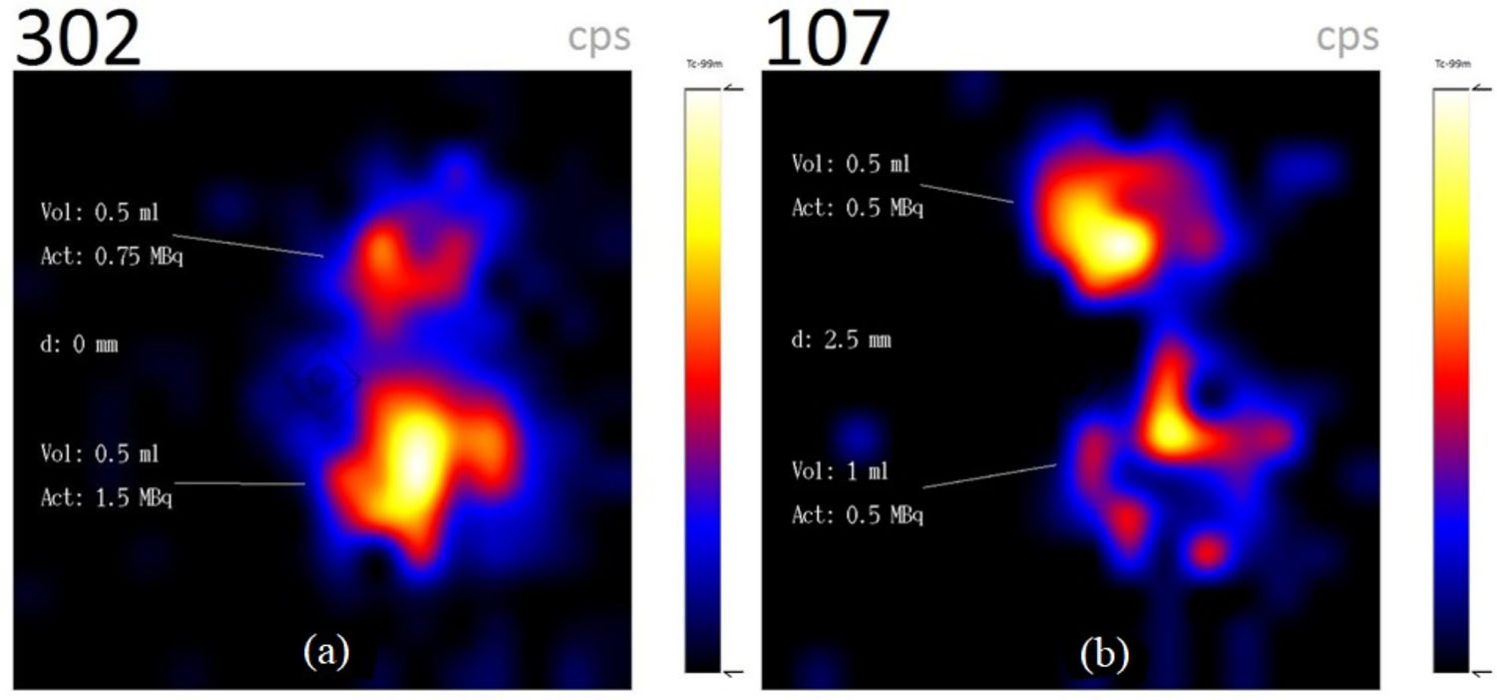
(a) Two spheres of the same volume but different activities and (b) two spheres with different volumes but the same activity.

The ratios of the total activities in Figure 6a,b (6/3.75 MBq) and total count rates (774/497 cps) are 1.60 and 1.56 respectively, thus confirming the good sensitivity of semiconductor detectors, especially for low energy γ‐rays.^14^ Similarly, two 0.5 mL spheres with activities of 1.5 and 0.75 MBq at an inter‐sphere distance of 0 mm mirroring two nodes of the same volume but different uptake activities are depicted in Figure 7a while Figure 7b shows a 0.5 and 1 mL sphere combination both with an activity of 0.5 MBq at an inter‐sphere distance of 2.5 mm. All spheres can be well differentiated.

### 3.3 Dual nuclide imaging

**FIGURE 8:**
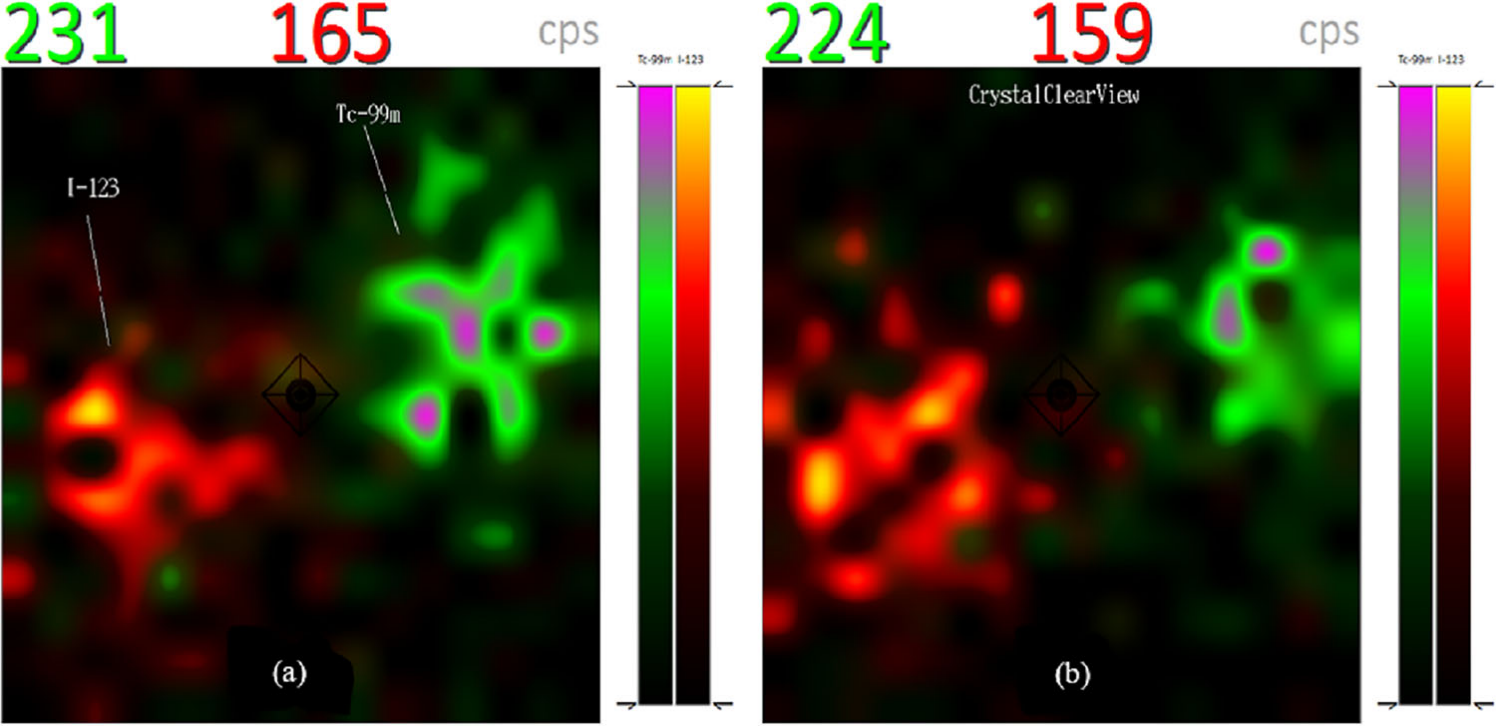
(a) Dual isotope imaging of two 1 mL spheres filled with equal activity of ^99m^Tc (green) and ^123^I (red) by disabling *CrystalClearView*. (b) same acquisition with *CrystalClearView* enabled.

Dual isotope imaging facilitates the simultaneous acquisition of two functional images without positioning error. The images of the two 1 mL spheres filled with equal activity (0.173 MBq) of ^99m^Tc and ^123^I, acquired with *CrystalClearView* disabled (default) and enabled are shown in Figure 8a,b respectively. Both imaging options show more ^99m^Tc count rates than ^123^I despite having the same activities.

**FIGURE 9:**
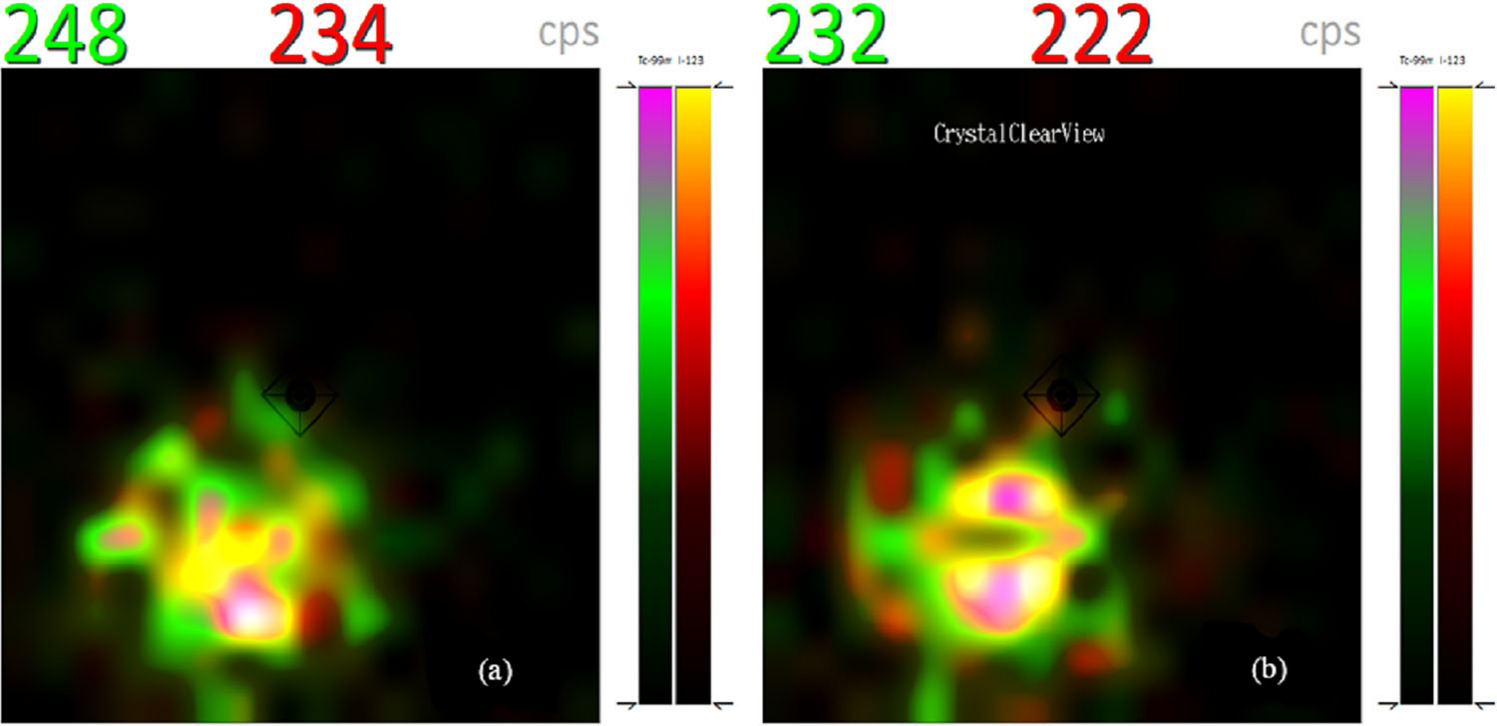
(a) Dual nuclide imaging with *CrystalClearView* disabled and (b) enabled show approximately equal count rates for ^99m^Tc (green) and ^123^I (red) despite an activity ratio of 1:2.

Figure 9a,b show images of the 1 mL sphere filled with a mixture of ^99m^Tc (0.06 MBq) and ^123^I (0.11 MBq) in default and enabled *CrystalClearView* respectively. Both nuclides have relatively the same count rates despite an activity ratio of 1:2.

### 3.4 Clinical studies

**FIGURE 10:**
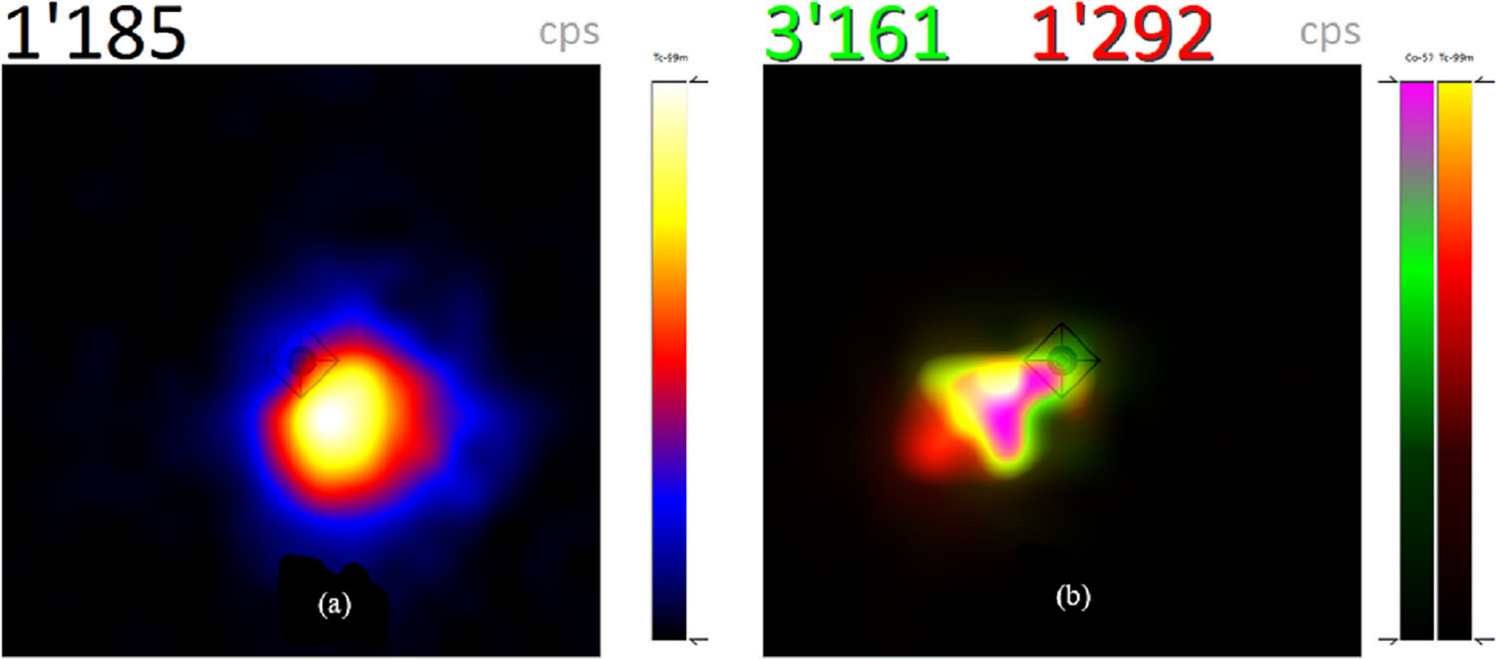
(a) Patient's localized SLN with CrystalCam, images acquired in ^99m^Tc energy window and (b) dual energy window of ^99m^Tc (red) and a ^57^Co (green) after marking SLN with a ^57^Co pen‐point marker on the patient's skin.

Figure 10 shows an SLN localized with CrystalCam in ^99m^Tc window (Figure 10a) and in ^99m^Tc and ^57^Co dual isotope windows (Figure 10b) acquired after the SLN in Figure 10a was marked with a ^57^Co pen‐point marker on the patient's skin. The ^99m^Tc (red) count rates increased slightly in Figure 10b compared to Figure 10a.

**FIGURE 11:**
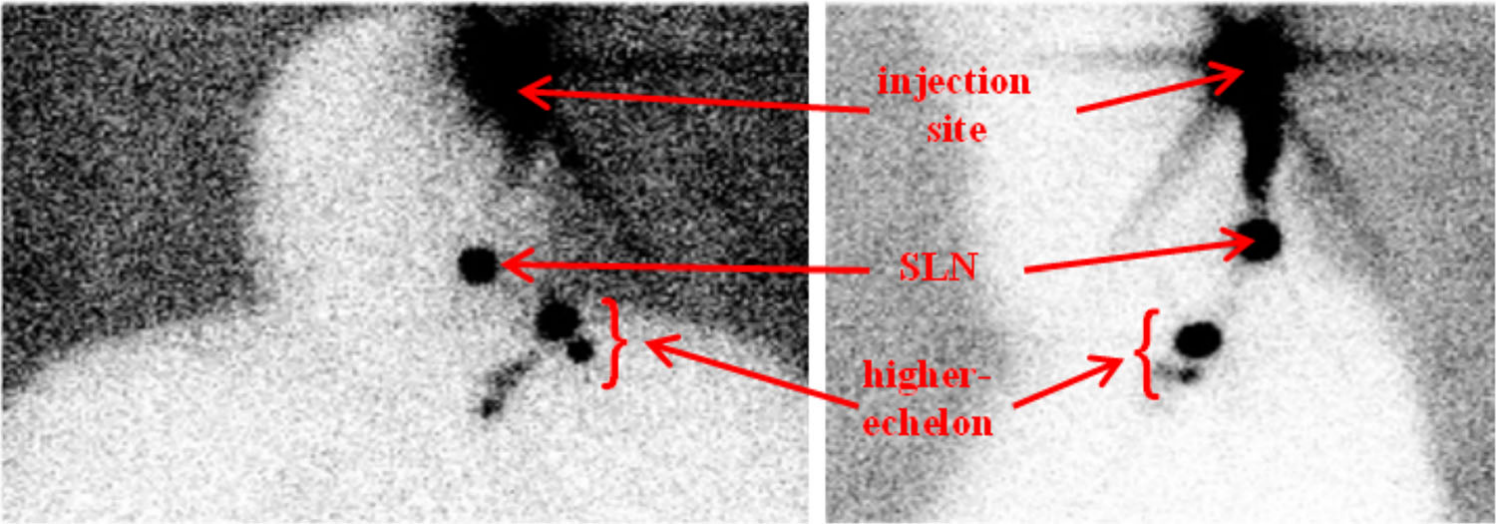
Patient's static planar images in the ventral (right) and lateral (left) projections with a ^57^Co flood source showing body contour, the injection site, SLN, and higher‐echelon nodes.

The static planar images of the head and neck region in the ventral and lateral left projections of the same patient acquired with a backlighting ^57^Co flood source used to create the patient's body contour, the injection site, SLN and higher‐echelon nodes (higher‐echelon nodes refer all labeled nodes that are not SLNs) are depicted in Figure 11.

For the 40 patients investigated in this study, SPECT/CT (the gold standard) localized a total of 151 lymph nodes consisting of 69 SLN and 82 higher‐echelon nodes. The location of the primary tumor (malignant melanoma) for the patients’ cohort in this study were in head/neck region (5), extremities (20), and trunk (15). In 3 of the 15 patients were the primary tumor was located at the trunk, the drainage of the tracer was in two different locations at the same time. CrystalCam missed 3 of the 8 SLNs detected by SPECT/CT in the 3 above mentioned patients. The other patients showed drainage in only 1 location. Lymphoscintigraphy and CrystalCam detected a total of 109 (72.19%) and 71 (47.02%) nodes respectively out of the 151 nodes detected by SPECT/CT. Table 4 shows the total lymph nodes detected by all three imaging modalities with respect to the body regions.

**TABLE 4:** The total lymph nodes detected by SPECT/CT, planar imaging, and CrystalCam with respect to body regions.

Body regions	Imaging modalities		
	SPECT/CT	Planar	CrystalCam
Head and neck	13	10	9
Axilla	55	44	35
Groin	83	60	31
Total	151	109	71

A total of 70 nodes were localized by both planar imaging and CrystalCam for which the total number of SLN and higher‐echelon nodes are 58 and 12 respectively. Both handheld camera and planar lymphoscintigraphy failed to localize a total of 41 nodes (8 SLNs and 33 higher‐echelon nodes) out of the 151 nodes detected by SPECT/CT.

A total of 69, 61, and 58 SLNs were detected by SPECT/CT, planar imaging, and CrystalCam respectively. The stratification of the localized SLNs with respect to body regions are depicted in Table 5. CrystalCam and planar imaging have 2 and 4 false positives respective.

**TABLE 5:** The total sentinel lymph nodes detected by SPECT/CT, planar imaging, and CrystalCam with respect to body regions. Undetected SLNs are entered in parenthesis.

Body regions	Imaging modalities		
	SPECT/CT	Planar	CrystalCam
Head/neck	10	8 (2)	8 (2)
Axilla	32	27 (5)	26 (6)
Groin	27	26 (1)	24 (3)
Total	69	61	58

For the head and neck region, both planar imaging and CrystalCam localized 8 SLNs and missed 2 SLNs of the 10 SLNs visualized by SPECT/CT. While CrystalCam and lymphoscintigraphy detected 80% of the total SLNs, both imaging systems detected and missed the same SLNs of the respective patients. The handheld gamma camera and planar scintigraphy, thus have a Cohen's kappa value of 1.00 and a 100% concordance rate for the detection of SLN in the head and neck region. Planar imaging and CrystalCam have 3 and 1 false positives respectively. For the axilla region, 32 SLNs were visualized by SPECT/CT of which 26 SLNs (81.25%) could be localized by CrystalCam while planar lymphoscintigraphy depicted 27 SLNs (84.38%).

Planar imaging depicted all the 26 SLNs detected by CrystalCam, thus scoring a Cohen's kappa value of 0.89 and a percentage agreement of 96.88% between CrystalCam and planar scintigraphy. For the McNemar *χ* ^2^ test, both imaging modalities have *p*‐value of 1.00.

For the groin, planar scintigraphy depicted 96.30% (26) of the total SLNs visualized by SPECT/CT (27), while the handheld gamma camera localized 88.89% (24) of the total SLNs. The McNemar *χ* ^2^ ‐test of planar imaging and CrystalCam shows a *p*‐value of 0.48, while the Cohen's kappa test shows a concordance rate of 92.59% and a Cohen's kappa value of 0.47. Both planar imaging and CrystalCam have 1 false positive SLN.

Table 6 shows the higher‐echelon nodes detected by planar lymphoscintigraphy, handheld gamma camera, and SPECT/CT.

**TABLE 6:** The total higher‐echelon nodes detected by SPECT/CT, planar imaging, and CrystalCam with respect to body regions. Undetected higher‐echelon nodes are entered in parenthesis.

Body regions	Imaging modality		
	SPECT/CT	Planar	CrystalCam
Head/neck	6	5 (1)	0 (6)
Axilla	24	14 (10)	8 (16)
Groin	52	29 (23)	5 (47)
Total	82	48	13

SPECT/CT visualized a total of 82 higher‐echelon nodes. Planar imaging with a conventional gamma camera localized 48 (58.54%) while handheld gamma camera detected only 13 (15.85%) of the total higher‐echelon nodes depicted by SPECT/CT.

In the head and neck region, CrystalCam was unable to detect a higher‐echelon node while planar imaging localized 5 (83.33%) of the 6 higher‐echelon nodes depicted by SPECT/CT. CrystalCam and planar lymphoscintigraphy could respectively localize 8 (33.33%) and 14 (58.33%) of the 24 higher‐echelon nodes depicted by SPECT/CT in the axilla. For the groin, planar scintigraphy depicted 55.77% (29) of the 52 higher‐echelon nodes visualized by SPECT/CT while CrystalCam could only localize 9.62% (5).

## 4 DISCUSSION

### 4.1 Sensitivity measurements

For any given volume, the respective positions of the 3 and 18 cm SDDs plots are at the top and bottom respectively, this further substantiate the inverse‐square‐law relationship between distance and radioactivity. In Table 3, the camera's count rate linearity or sensitivity reduces with increasing SDD as expected and the *R* ^2^ of approximately 1 signifies an excellent linear relationship between mean count rates and source activities at all SDDs. The very minimal effect of scattering on the measured count rates of the 16 mL sphere compared to the 0.5 mL at an SDD of 18 cm observed in Figure 5 is largely due to the high scattered rejection of the semi‐conductor detectors of CrystalCam. Abe et al.^15^ measured a sensitivity of 476.5 cps/MBq for their hand‐held semiconductor‐based gamma camera, eZ SCOPE while Knoll et al.^11^ measured a CrystalCam sensitivity of 554 cps/MBq. Although both studies did not specify the SDDs, these sensitivities correspond to the measured sensitivities of this study at an SDD of approximately 5 and 6 cm respectively. SLN usually encounter in melanoma are superficial at distances below 3 cm. The design of the Jaszczak phantom prevents measurements below 3 cm in this study. The handheld camera sensitivity for distances below 3 cm or any arbitrary distance can be extrapolated from the results in Table 3. The sensitivity measurement results affirm CrystalCam's ability to accurately quantify the uptake activity of deep‐seated, low‐contrast target organs especially in the derivation of time‐activity curves during nuclear medicine therapies.

### 4.2 Resolution measurements

Accurate localization of SLN close to an injection site is very challenging, especially when there's a slow rate of radiotracer migration from the injection site to SLNs due to the high radioactive background. In Figure 6a, the low activity 0.5 mL sphere (node) with activity 0.75 MBq is distinguished from the 1 mL sphere (injection site) with 3 MBq activity.

Usually, if there are more than one SLN, high SLN uptake with subsequent washout of radiopharmaceutical to the higher‐echelon nodes results in SLNs with larger volumes having higher radiotracer uptake compared to smaller SLNs provided there are no obstructions in the lymphatic drainage system and there's enough injected activity that is, larger foci having a higher target/background ratio. This clinical scenario is depicted by the spheres in *Group A*. Similarly, two nodes of the same volume will have the same uptake activity as illustrated by the two 1 mL spheres in Figure 6b.

SLNs of different sizes occasionally have the same uptake as illustrated by the spheres in *Group B*. This clinical scenario is observed in massively metastatic SLN, where extensive replacement of the normal tissue with tumor cells may result in; two nodes of the same volume having different uptake activity due rerouting of lymph fluid to a “neo–sentinel node” that does not harbor metastasis (false‐negative)^4^, ^16^ as shown in Figure 7a, or two nodes with different volumes having the same activity as a result of the massive tumor invasion of the SLN completely obstructing the lymph flow, preventing tracers from accumulating in the sentinel node and thus preventing its identification as demonstrated in Figure 7b.

All three sphere combinations of Figures 6, 7, and 7b were clearly differentiated when they were touching each other (*d* = 0 mm) while the spheres are completely separated at inter‐sphere distances greater than 3 mm due to the high energy resolution and scattered rejection of the semiconductor detectors compared to scintillation detectors, generally using a NaI(TI) crystal.^17^, ^18^

Spheres touching each other (*d* = 0 mm) were also easily differentiated due to partial volume effect of the Perspex walls of the spheres thus, generating a scintigraphic cold region between the active regions.

The observance of activity ratio of about 1:100 for injection sites and nodes proximity compared to the 1:4 activity ratio (0.75:3 MBq) in this study necessitate further studies to ascertain the handheld camera's ability to differentiate a node close to an injection site.

### 4.3 Dual nuclide imaging

The photo peaks of both nuclides are closed to each other resulting to overlapping energy windows; 140 keV ± 7.5% (129.5–150.5 keV) for ^99m^Tc and 159 keV ± 7.5% (141.1–170.9 keV) for ^123^I.

Although both nuclides have the same activity, ^99m^Tc has more count rates in the default settings (Figure 8a) since the entire Compton continuum (low scattered photons) of ^123^I falls into the ^99m^T energy window. Enabling *CrystalClearView* only reduces the count rates of ^99m^T and ^123^I by 7 and 6 cps respectively (Figure 8b). In Figure 9a, ^99m^Tc and ^123^I have almost the same count rates despite an activity ratio of 1:2 implying about 50% of ^123^I‐photons fall into the ^99m^Tc energy window. Enabling *CrystalClearView* only reduces the count rates of ^99m^Tc and ^123^I by 16 and 12 cps respectively (Figure 9b).

The difference in count rates with *CrystalClearView* disabled (Figures 8a and 9a) and enabled (Figures 8b and 9b) are statistically insignificant since the average background count rates of the room was 27 cps and the fact that *CrystalClearView* also reduces ^123^I at an equal ratio as ^99m^Tc implies the *CrystalClearView* tool does not yield accurate quantitative results during dual isotope imaging of two nuclides with overlapping energy window as stipulated by the manufacturer.^12^ Although there is currently no technique using dual isotopes in the localization of SLN in melanoma, the need to mark the detected SLN on the patient's skin with a ^57^Co pen‐point marker in routine clinical practice; necessitate the investigation of the effect of *CrystalClearView* in dual nuclide imaging due the overlapping energy windows of the ^99m^Tc and ^57^Co.

The ^57^Co energy window (112.9−131.2 keV) overlaps with the ^99m^T (129.5–150.5 keV) by about 2 keV which corresponds to the upper end of the ^57^Co energy spectrum which is usually photon deficient. The few high energy ^57^Co‐photons falling into ^99m^T energy window are responsible the slight increase in ^99m^Tc count rates from 1185 cps in Figure 10a (without ^57^Co) to 1293 cps 10b (with ^57^Co). The *CrystalClearView* tool was disabled during dual nuclide imaging of ^57^Co and ^99m^T due to its inability to correct the count rates for overlapping energy window.

### 4.4 Clinical studies

The image quality acquired by CrystalCam compared to conventional planar imaging is due to motion artefacts since the CrystalCam moved at arbitrary speed during imaging. The handheld camera could not be held at a fixed SDD due to the different body regions.

Planar lymphoscintigraphy and CrystalCam show a perfect agreement for the localized SLN in the head and neck region as illustrated in Table 5. This is mostly due to the increased flexibility in handling CrystalCam in this region. CrystalCam could easily be held at different angles and distances from the skin surface. The two SLNs missed by both planar imaging and CrystalCam may be due to superimposed SLNs which are difficult to differentiate using 2D imaging modalities.

In the axilla region, CrystalCam and planar imaging statistically exhibit an almost perfect agreement with CrystalCam missing only 1 SLN that was localized by planar imaging. The unidentified SLN can be associated with the small FOV of the handheld gamma camera compared to conventional gamma camera. Planar imaging and the handheld camera show a moderate agreement in localizing SLNs in the groin.

The false positives SLNs identified by planar imaging (4) and CrystalCam (2) may be due to the challenges associated with 2D imaging systems to differentiate an injection site or lymphatic channels from SLNs because of their poor resolution compared to SPECT/CT imaging. Shielding the injection site would have help to localize SLNs situated close to the injection site and reduce the dead time of the handheld gamma camera, thus providing the possibility of measuring with a higher acquisition time at short SDD.

The 95.65% concordance rate for the total SLN detected in head and neck, axilla, and groin using planar scintigraphy with the conventional gamma camera and the handheld semi‐conductor gamma camera implies that; in 1 of 20 patients, an SLN was not visualized by the handheld camera compared to the conventional planar imaging system. Although the identification rate of SLN with the conventional planar imaging is better the handheld gamma camera, there is no significant statistical difference between both camera system in SLN localization.

The solid‐state detector handheld gamma camera was unable to localize a higher‐echelon node in the head and neck region and only 5 in the groin compared to the 5 and 29 higher‐echelon nodes detected by planar imaging in the head and neck region and groin respectively.

The poorer image quality acquired by CrystalCam for higher‐echelon nodes visualization with respect to planar imaging (*p*‐value = 2.3E‐8), may be due to the larger FOV of conventional gamma camera compared to the handheld camera, since the CrystalCam physician does not know where to position the camera during higher‐echelon nodes detection. The short acquisition time of the CrystalCam in this study compared to planar lymphoscintigraphy is also a reason for the poor higher‐echelon nodes localization.

Further investigation with CrystalCam held about 10 cm above the patient's skin to increase the FOV including longer acquisition time during higher‐echelon nodes visualization are required. An optimum SDD is paramount since, holding CrystalCam at larger distances above the skin will also reduce the sensitivity of the handheld camera.

The satisfactory detection results of semiconductor detection system CrystalCam in both the phantom and clinical studies compared to conventional gamma camera imaging using NaI(TI) scintillation crystal connected to a photo multiplier tube are due to the high energy resolution and scattered rejection of solid‐state detectors The semiconductor detector also gives direct counting of the incident photon resulting to a short acquisition time compared to the large signal loss due to light moving from the scintillator to the photo multiplier tube.^17^, ^18^

## 5 CONCLUSION

The handheld gamma camera CrystalCam was specifically designed for SLN localization. In this study, CrystalCam was used for preoperative SLN identification in malignant melanoma compared to planar imaging with a conventional gamma camera and SPECT/CT imaging.

The handheld camera was statistically inferior to the planar imaging with a conventional gamma camera in the localization of higher‐echelon nodes due to its small FOV and the short acquisition time used in this work. The results of this study show that planar scintigraphy with conventional gamma camera remains the preferred modality for higher‐echelon nodes localization.

Although the detected SLNs with planar lymphoscintigraphy with conventional gamma camera in the axilla and groin regions are more than the handheld gamma camera, the difference of the total SLNs localized by both imaging system are not statistically significant.

The handheld gamma camera CrystalCam is thus a reliable instrument for localizing SLN in surgical centers without an on‐site nuclear medicine department. In nuclear medicine centers, the handheld camera allows the physician to perform pre‐ and intra‐operative lymphoscintigraphies in a separate room, hence freeing up the conventional gamma camera for other diagnostic procedures.

## AUTHOR CONTRIBUTIONS

Isong Assam: Conceptualization, data acquisition, analysis or interpretation of data, and drafting of the manuscript. Simon P. Dierck: Data acquisition, analysis or interpretation of data, and drafting of the manuscript. Yi Zhao and Michael Jüptner: Critical revision of the manuscript for important intellectual content. Frank‐André Siebert: Critical revision of the manuscript for important intellectual content and final approval of the version to be published. Maaz Zuhayra: Conceptualization, critical revision of the manuscript for important intellectual content, and final approval of the version to be published. Ulf Lützen: Initial idea, conceptualization, interpretation of data, critical revision of the manuscript for important intellectual content, and final approval of the version to be published.

## CONFLICT OF INTEREST STATEMENT

The authors declare no conflicts of interest.
